# Super-long life time for 2D cyclotron spin-flip excitons

**DOI:** 10.1038/srep10354

**Published:** 2015-05-19

**Authors:** L. V. Kulik, A. V. Gorbunov, A. S. Zhuravlev, V. B. Timofeev, S. Dickmann, I. V. Kukushkin

**Affiliations:** 1 Institute of Solid State Physics, Russian Academy of Sciences, Chernogolovka, 142432 Russia

## Abstract

An experimental technique for the indirect manipulation and detection of electron spins entangled in two-dimensional magnetoexcitons has been developed. The kinetics of the spin relaxation has been investigated. Photoexcited spin-magnetoexcitons were found to exhibit extremely slow relaxation in specific quantum Hall systems, fabricated in high mobility GaAs/AlGaAs structures; namely, the relaxation time reaches values over one hundred microseconds. A qualitative explanation of this spin-relaxation kinetics is presented. Its temperature and magnetic field dependencies are discussed within the available theoretical framework.

The problem of spin relaxation in solids remains the main focus of research related to spintronics and the search for long-lived non-equilibrium spin systems that could be promising for the realization of quantum calculations. A long lifetime of the excited quantum states in two-level systems is an essential condition for the practical realization of quantum information bits (qubits). The majority of research aimed at creating solid-state qubits until now has been focused on issues of electron spin relaxation in quantum-confined structures: quantum dots^1^,^2^,^3^, impurity centers^4^, and strongly correlated quasi-one-dimensional systems related to chiral Hall currents^5^,^6^. We present the results of our research into super-long spin relaxation times for a translation-invariant (microscopically delocalized) quantum object, the cyclotron spin-flip exciton (CSFE), which can be manipulated using the photoexcitation of two-dimensional (2D) electrons.

It is known that exciton states in metal systems, such as a 2D electron gas, are unstable^7^. However, 2D metal becomes an insulator for integer quantum Hall states in quantizing magnetic fields at temperatures significantly lower than the cyclotron energy. Neutral excitations in quantum Hall insulators are magnetoexcitons (bosons) by analogy with the magnetoexcitons in 3D semiconductor systems^8^. Indeed, the analogy applies if a cyclotron, Zeeman or Coulomb gap replaces the forbidden energy gap in semiconductors. The occurrence of a bosonic component in a correlated fermion system makes the quantum Hall states a potential candidate for the formation of macroscopic, nonequilibrium condensates, demonstrating superfluidity as suggested in^9^.

One of the simplest realizations is the magnetoexciton induced by an electron promoted from the zeroth to the first Landau level in a spin-unpolarized quantum Hall system at a filling factor of *v* = 2. The excitation spectrum exhibits two types of inter-Landau-level magnetoexcitons: a spin-singlet with a total spin of *S* = 0 possessing an energy equal to the cyclotron gap (according to the Kohn theorem^10^); and a CSFE spin-triplet with a total spin of *S* = 1 and spin projections of −1, 0, *and* +1 along the magnetic field axis^11^. The singlet magnetoexciton represents the spinless magnetoplasma mode, and its relaxation is related to the transition of an electron from the first to the zeroth Landau level. This process may occur via dipole cyclotron radiation, and this radiative mechanism is the dominant relaxation channel in this case^12^. In other words, the spin-singlet exciton is a `bright exciton'. In contrast to the spin-singlet, the spin-triplet exciton is not radiatively active due to electron spin conservation. Thus, the spin-triplet exciton is a *dark* exciton. Additionally, there are no symmetry restrictions similar to the Kohn theorem for the spin-triplet exciton, and its energy is reduced relative to the cyclotron energy due to the Coulomb interaction^13^,^14^. An excitation of the CSFE component *S* = 1, *S*_*z*_ = − 1 will change the spin state of the electron system (see diagram in Fig. 1), therefore the CSFE relaxation is the spin relaxation for the entire electron system.

The optical excitation of electrons to higher energy states can be used to produce a significant spin-triplet exciton density. The relaxation of the spin-triplet exciton to the ground state accompanied by electron spin flip is expected to be a long-term process^15^. If the electron system temperature is much lower than the energy gap between the spin-singlet and spin-triplet excitons, the latter has no optical decay channels, and the only nonradiative channel for spin-triplet exciton relaxation to ground state is the emission of high-frequency acoustic phonons in the presence of spin-orbit coupling. The present paper aims at generating the *S* = 1, *S*_*z*_ = − 1 exciton ensemble and investigates the relaxation of this ensemble to the ground state.

A high–electron mobility of the 2D system is essential for spin-triplet exciton observation. Therefore, we considered high-quality heterostructures with symmetrically doped GaAs/AlGaAs single quantum wells. Optical measurements were made using two continuous wave tunable lasers (Fig. 1, top panel)): one for resonant excitation of the electron system and the other for recording the spectra of resonance reflectance (RR), photoluminescence (PL), and inelastic light scattering (ILS). Because detailed spectral reflectance profile analyses in doped systems can be fairly complicated^16^, it can be postulated that for the *v* = 2 quantum Hall insulator, the reflectance signal of a laser tuned to the transition from the zeroth Landau level of heavy valence holes to the zeroth Landau level of conductance band electrons will not be observed, because all of the electron states are occupied. With a supplementary pump that promotes the electrons to higher Landau levels, one can expect the formation of dark excitons, *S* = 1, *S_z_* = − 1, as they occupy the lowest energy excited state in which the pumped carriers relax (considering the negative value of the electron g-factor in GaAs, *S* = *S_z_* = 1). This process should manifest itself as a decrease in the unoccupied states on the first electron Landau level and the occurrence of empty states (vacancies) on the zeroth electron Landau level. The respective change in RR is called the photoinduced resonance reflection (PRR).

The PRR spectrum should have two peaks: a positive peak in the transition region from the zeroth Landau level of heavy holes to the upper spin sublevel of the zeroth electron Landau level and a negative peak in the transition region from the first valence heavy-hole Landau level to the first electron Landau level. The positive peak is responsible for the formation of vacancies on the upper spin sublevel of the zeroth electron Landau level, while the negative peak is responsible for the decrease in the number of vacancies on the lower spin sublevel of the first electron Landau level. Thus, we propose a photoinduced resonance reflectance technique for indirectly testing the presence of dark excitons through optically allowed transitions from the valence band to the conductance band.

## Results

**Figure 1:**
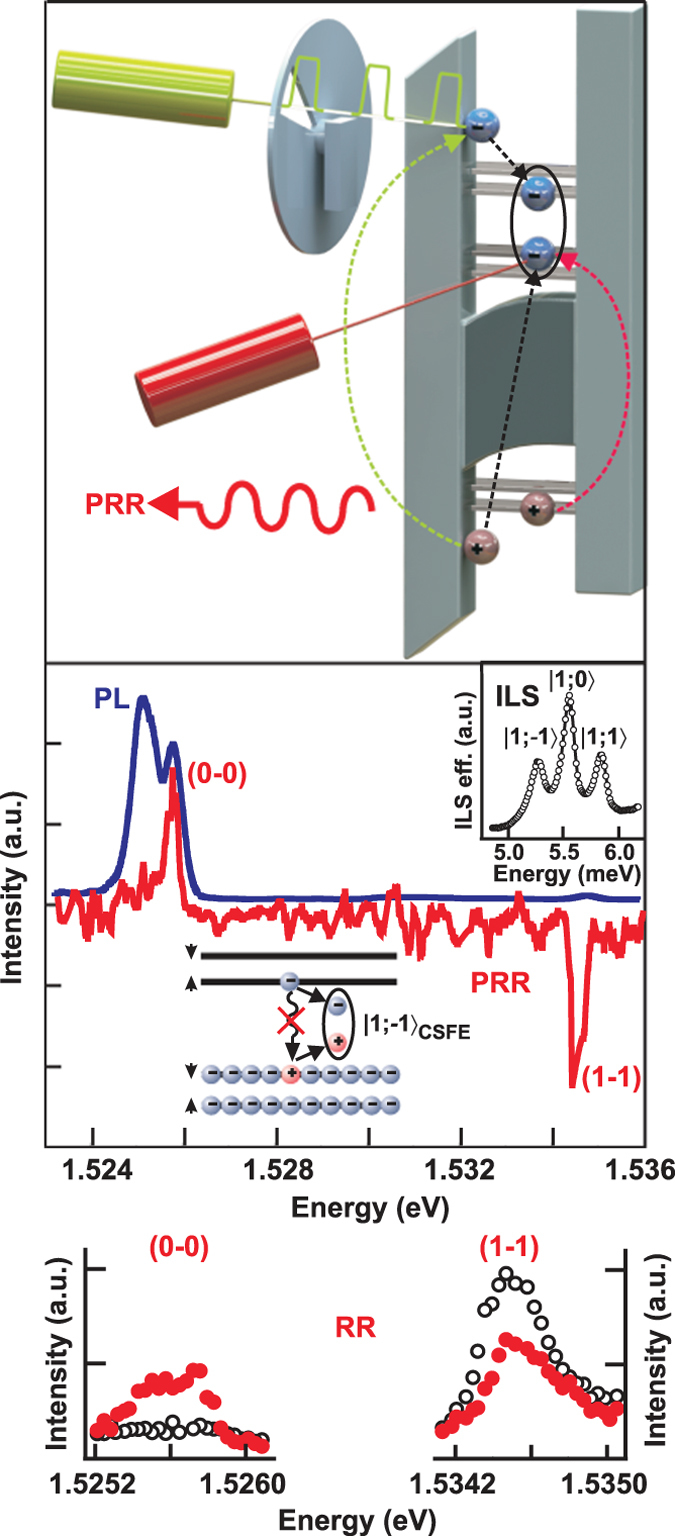
Photoluminescence (PL) and photoinduced resonant reflectance (PRR) spectra taken at *v* = 2 in a 17 nm quantum well (the dark mobility is 5 × 10^6^ cm^2^/Vs, the electron concentration is 2.4 × 10^11^ cm^−2^) for the magnetic field *H* = 5 T normal to the quantum well and bath temperature of 0.45 K. The diagram shows how the lowest energy excited state forms. The inset demonstrates the inelastic light scattering (ILS) spectrum of the cyclotron spin-flip exciton taken at similar experimental conditions (perpendicular magnetic field and temperature) with a 5 T extra–parallel magnetic field to enhance the Zeeman energy splitting above the experimental spectral resolution. Detailed resonant reflectance (RR) spectra with (closed dots) and without (open dots) extra pumping are shown in the bottom. (top) Scheme of the experimental setup.

The central panel of Fig. 1 shows the representative experimental PL and PRR spectra taken at *v* = 2. The detailed RR spectra with and without extra pumping are presented in the bottom of Fig. 1. The PRR spectrum looks just as it was anticipated: a positive peak at the (0-0) transition line (the first figure refers to the valence heavy-hole Landau level and the second to the electron Landau level) and a negative peak in the region of (1-1) transition. This pattern is observed in the experiment, which is related to the formation of low energy magnetoexcitons composed of electrons on the first Landau level and vacancies on the zeroth electron Landau level (see the diagram in Fig. 1). Despite the inactivity of cyclotron spin-triplet excitons with respect to radiative decay, their actual existence is established by ILS, and the shift of the central triplet component *S* = 1, *S*_z _= 0, from the cyclotron energy is used to determine the energy gap between the spin-triplet and spin-singlet excitons (Fig. 1, inset).

**Figure 2:**
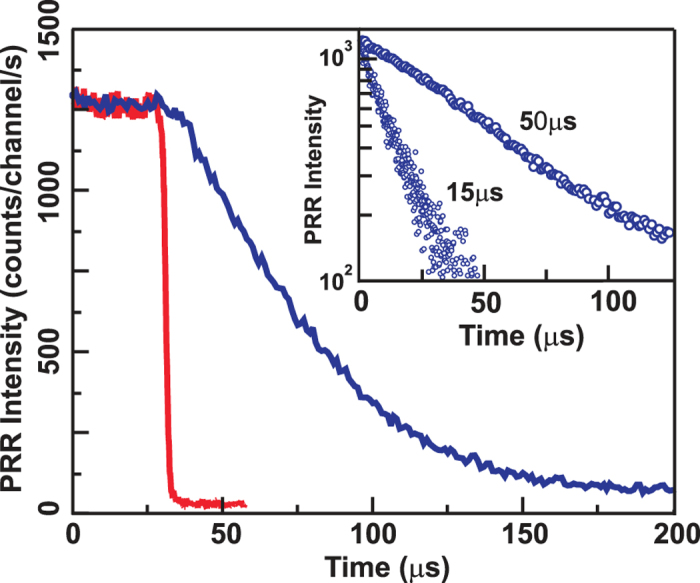
Example of a photoinduced resonant reflectance (PRR) decay curve with the apparatus function of the chopper (red). The inset shows two decay kinetics for quantum wells of 35 (longer) and 17 nm (shorter) width taken at *v* = 2 at 4 T perpendicular magnetic field and bath temperature of 0.45 K. The electron concentration in the quantum wells is 2.0 × 10^11^ cm^-2^, the mobilities are 1.5 × 10^7^ cm^2^/Vs and 5 × 10^6^ cm^2^/Vs, respectively.

**Figure 3:**
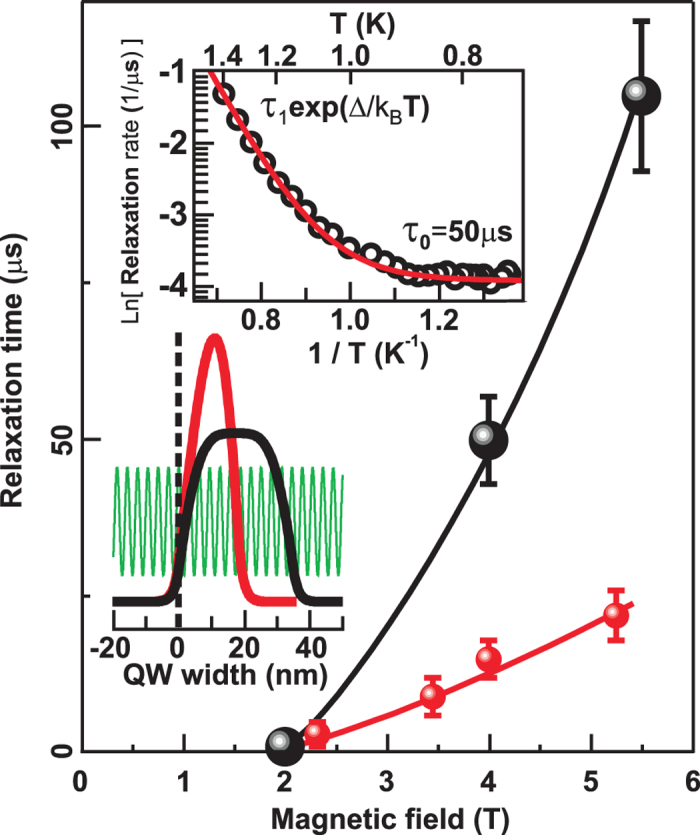
An assembled graph of low temperature relaxation constant *τ*_0_ as a function of magnetic field. The small points denote the magnetic field dependence for quantum wells of 17 nm widths. The large points denote the magnetic field dependence for quantum wells of 35 nm widths. The curves are drawn for clarity. Top inset: relaxation rate vs. temperature in logarithmic (ln) scale taken at *v *= 2 in the 35 nm quantum well at 4 T magnetic field (dots) with a fitting using two relaxation mechanisms, activated and temperature-independent (line).The diagram shows the electron size quantized envelopes for two quantum wells of 17 and 35 nm widths and a schematic wave function of the phonon emitted to illustrate an incommensurability between the phonon wave length and the extent of the electron wave function in the growth direction of the quantum wells.

The pumping laser emission was modulated periodically to measure the magnetoexciton relaxation kinetics (Fig. 2). The PRR decay curve on the (0-0) transition line appears to be extremely long. The PRR signal decays over tens of microseconds upon switching off the resonant pumping (Fig. 2). The PRR (1-1) signal in turn increases over the same time, which points to common relaxation dynamics for the exciton states formed by electrons on the first and by holes on the zeroth Landau level. The common specific feature of the observed relaxation kinetics is their temperature dependence. The temperature dependence for the spin relaxation rate is exponential at high temperatures above 1 K, with a characteristic relaxation time (*τ*_1_) on the order of 1 nanosecond and an energy gap (Δ) of approximately 11 K (Fig. 3). This is indicative of an optically activated relaxation channel consisting of an electron spin-flip due to spin-orbit coupling, increasing the exciton energy to the cyclotron energy, and emitting a photon with the cyclotron energy^12^. We have measured the Coulomb term independently for the sample in question, and it amounts to 0.54 meV; hence, the Coulomb gap is 6.3 K. We should add the Zeeman term for electrons to this quantity (an electron should rotate its spin when relaxing to the ground state). This adds 1.2 K at 4 T to the Coulomb term. We have a total of 7.5 K, which is somewhat less than the obtained 11 K.

The kinetics are no longer temperature dependent at lower temperatures, i.e., the relaxation mechanism changes. Temperature-independent relaxation implies a non-activated mechanism for the exciton decay. The most intuitive relaxation channel could be represented by the decay into short-wave acoustic phonons described in^15^. The relaxation time *τ*_0_ is predicted to depend superlinearly on the extent of the electron wave function in the direction of quantum well growth (in the *z*-direction). To estimate this effect, two types of quantum wells, 17 and 35 nm wide, with nearly equal electron densities were chosen so that the half-widths of their electron envelope wave functions had approximately a two-fold difference. Fig. 3 confirms qualitatively the main prediction of^15^. This Figure supports also another prediction of^15^: the relaxation rate falls with magnetic field, as the emission of hard phonons with large frequencies (~*ω*_c_) is difficult due to reduction of the electron-phonon coupling.

## Discussion

We will now discuss the features of the experimental data obtained. (i) The CSFE relaxation is extremely slow, which, in principle, corresponds to the general theoretical prediction. However, the characteristic times (Fig. 3) still differ significantly from the 7 milliseconds calculated for CSFE annihilation^15^. (ii) With a growing magnetic field, i.e., with an increasing cyclotron gap, the relaxation becomes appreciably slower (Fig. 3), which excludes the radiation mechanism (the latter should, on the contrary, be enhanced owing to the dipole irradiation law ~*ω*^3^_c_) and suggests a phonon-emission relaxation process. (iii) Relaxation in a wider quantum well occurs more slowly (see the diagram in Fig. 3), which is also a qualitative indication of a phonon-emission relaxation channel (the larger the extent of the electron wave function in the *z*-direction, the smaller the effective electron coupling to a short-wave acoustic phonon). (iv) Relaxation does not depend on the temperature below a characteristic temperature, which is consistent with an acoustic phonon relaxation mechanism. (v) Finally, within our experimental accuracy, relaxation occurs exponentially with time, i.e., the relaxation rate is independent of the CSFE concentration.

The last experimental observation contradicts the CSFE–CSFE scattering relaxation scenario^15^, which assumes a single-phonon emission resulting in the annihilation of one of the two excitons, and therefore leading to a non-exponential time dependence of the relaxation rate. Additionally, the fact that the observed relaxation times are much shorter than those predicted forces us to correct the existing theoretical assumptions. Recall that the extremely long CSFE relaxation is essentially due to the small factor 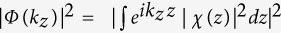, where *χ*(z) is the 2D electron size-quantized wave function and 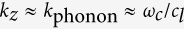 (*ω*_c_ to denote the cyclotron frequency, and 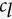 is the longitudinal sound velocity). Yet, the energy release via phonon emission could be a more complicated process than previously thought, i.e., two- or even multi-phonon emission channels may occur.

Let us consider, for example, the *two-phonon* emission mechanism. It may be realized as a *single-exciton* annihilation process representing a transition from the initial CSFE state to the final state with two longitudinal 3D phonons with wave vectors *k*_*1*_*=*(*−q,k*_*z1*_) and *k*_*2*_ *=* (*−q,k*_*z2*_). In-plane wave-vector components with *q~*1/*l*_*B*_ (*l*_*B*_ is the magnetic length) provide the most effective electron–phonon coupling in a strong perpendicular magnetic field, whereas due to energy conservation, the *z*-components must satisfy the condition 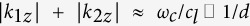 (*d* is the characteristic extent of the electron wave function in the *z*-direction). For two-phonon emission processes, the small vertex of the electron–phonon interaction is perturbatively accounted for within the second-order approximation (whereas the spin–orbit coupling is still considered to the first order). However, the two-phonon relaxation channel could be faster than the single-phonon because (i) as the emitted phonon phase volume becomes larger, the momentum conservation law allows phonons to have anti-parallel components *q~*1/*l*_*B*_, effectively contributing to the relaxation; (ii) the relevant two-phonon transition matrix element is enhanced because it is determined by numerous intermediate virtual-exciton states corresponding to an electron promoted to various Landau levels with numbers *n* ≥ 1; (iii) finally, the product 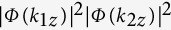 enters the final result for the relaxation rate rather than the factor 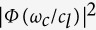. Appropriate analysis reveals that the most effective relaxation is realized via the emission of phonons with 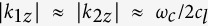, and the factor 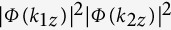 may be larger than 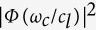 as a result. Similar arguments are valid for a multi-phonon process contributing to the single-exciton annihilation. Therefore, it is no wonder that the averaged single-exciton relaxation matrix element may exceed the double-exciton relaxation matrix elements theoretically studied in^15^.

In conclusion, we have created an ensemble of 2D translation-invariant cyclotron spin-flip excitons and investigated its relaxation kinetics. The spin-excitons (spin waves) associated with a spin flip within a single Landau level have been known thus far to possess the longest relaxation times of aproximately 100 ns measured among translation-invariant spin excitations in 2D-electron systems^17^. The studied spin-triplet exciton associated with electron spin flip and the simultaneous change of cyclotron quantum number now relaxes to the ground state over tens or even *hundreds of microseconds*. The unprecedentedly large relaxation times for excitations in the translation-invariant system are partly due to a specific Coulomb confinement in the conjugate *K*-space that blocks some relaxation channels and thereby prevents excited electrons from backward spin flipping. Coulomb confinement is somewhat similar to spatial 3D confinement forming quantum dots and also resulting in the slowdown of single-electron spin relaxation^2^. Owing to such long relaxation times, optical pumping can be used to generate cyclotron spin-flip excitons with densities sufficient to reveal their collective Bose properties. In case of successful realization of this scenario the long living cyclotron spin-flip exciton ensemble may appear as a second example, after electron-electron bilayers^18^, of a dense system of Bose particles in degenerate 2D Fermi gas, which may demonstrate Bose-Einstein condensation effects.

## Methods

### Samples

We explored two sets of high-quality heterostructures (the dark mobility in the range of 5–20 × 10^6^ cm^2^/Vs) with symmetrically doped GaAs/AlGaAs single quantum wells of two widths: 17 and 35 nm. Every set included a number of samples with different electron concentrations in the 2D channel ranging from 5 × 10^10^ to 2.5 × 10^11^ cm^-2^. Measurement of every sample characterized by its unique electron concentration gave us one point in the graph of spin relaxation time versus magnetic field.

### Spectral measurements

The sample of size of approximately 3x3 mm^2^ was placed into a pumped cryostat with liquid ^3^He, which in turn was placed into a ^4^He cryostat with a superconducting solenoid. The setup allowed measurements at the bath temperature down to 0.45 K and at the magnetic field up to 14 T. Optical measurements were made using the dual-fiber technique with the use of multimode quartz glass optical fiber with a core diameter of 400 *μ*m and numerical aperture N. A. of 0.39. One fiber was used for photoexcitation, and a second for collecting the emission from the sample and transferring it onto the entrance slit of a grating spectrometer equipped with a CCD camera cooled by liquid nitrogen. Two continuous wave tunable lasers with narrow spectral widths of emission lines (20 and 5 MHz) were employed as optical sources, which enabled us to use one of the lasers for resonant excitation of the electron system and the other for recording spectra of resonance reflectance, photoluminescence, and inelastic light scattering.

The experimental geometry was chosen when measuring the resonance reflectance spectra so that the specularly reflected beam axis coincided with the receiving fiber axis at an angle of incidence of approximately 10°. The contribution of the sample surface reflection was suppressed using crossed linear polarizers set between the sample and the ends of the pumping and collecting fibers. One of the lasers was used as an optical pump of the system via the excitation of the electrons to high energy Landau levels, *n* > 1, in the experimental studies of photo-induced reflection. The pumping laser intensity was limited to a power below 0.3 mW to minimize heating effects. An emission from a probing laser, which was weaker by an order of magnitude, was coupled into the same waveguide. The resonance reflectance spectrum was obtained by scanning the emission wavelength of the probing laser and registering the laser line intensity using the spectrometer with the CCD camera. The photoinduced resonant reflection was obtained as the difference in the resonance reflectance spectra with the resonance pump switched on and off.

### Kinetic measurements

The pumping laser emission was modulated periodically using a mechanical chopper (a rotating disk with a radial slit), in the experiment on photoinduced reflection kinetics. The modulation period was approximately 11 ms, and the pump pulse rise/decay time was less than 2 *μ*s. The laser beam was focused onto the chopper disk surface by a microscope objective to shorten the pump pulse front. The excitation energy of the probing laser was set to the maximum/minimum of the photoinduced resonance reflectance spectrum to record the decay/rise curve, respectively. The probing laser emission reflected from the sample was passed through a tunable narrow-band interference filter of 1.1 nm spectral width to cut off the pumping laser light and then focused on an avalanche photodiode operating in the photon counting regime. A gated photon counter was used to accumulate the photoinduced resonant reflection signal as a function of the time delay from the pump cut-off event to obtain a photoinduced resonant reflection decay curve with an acceptable signal-to-noise ratio.

## Author Contributions

L.V.K., A.V.G., A.S.Z., V.B.T., and I.V.K. planned experiments. L.V.K., A.V.G., and A.S.Z. performed the experiments and analyzed data. L.V.K., A.V.G., and V.B.T. wrote the main manuscript text. S.D. wrote the theoretical part of the manuscript. L.V.K. and A.S.Z. prepared figures. All authors have given approval to the final version of the manuscript.

## Additional Information

**How to cite this article**: Kulik, L.V. *et al.* Super-long life time for 2D cyclotron spin-flip excitons. *Sci. Rep.* **5**, 10354; doi: 10.1038/srep10354 (2015).
